# Concerns and recommendations: antiplatelet agents and cancer prevention in non-alcoholic fatty liver disease

**DOI:** 10.3389/fonc.2025.1607955

**Published:** 2025-07-14

**Authors:** Hong Tham Pham, Kim-Huong Truong-Nguyen, Minh-Hoang Tran

**Affiliations:** ^1^ Faculty of Pharmacy, Nguyen Tat Thanh University, Ho Chi Minh, Vietnam; ^2^ Department of Pharmacy, Nhan Dan Gia Dinh Hospital, Ho Chi Minh, Vietnam; ^3^ NTT Hi-Tech Institute, Nguyen Tat Thanh University, Ho Chi Minh, Vietnam

**Keywords:** antiplatelet agent, cancer, non-alcoholic fatty liver disease, confounding, bias

## 1 Introduction

The study entitled “The chemoprotective effect of anti-platelet agents on cancer incidence in people with non-alcoholic fatty liver disease (NAFLD): a retrospective cohort study,” published in the BMC Medicine (1), provides novel insights into the potential link between antiplatelet agents and cancer incidence in NAFLD, underscoring the importance of further research. Nevertheless, we have several concerns regarding the study’s design, statistical approach, and interpretation of findings that were not adequately addressed. These issues are likely to challenge other investigators if not being aware of. We provide some recommendations to strengthen the robustness of future studies and identify the underlying protective mechanisms of antiplatelet agents outside cardiovascular areas.

## 2 Concerns and recommendations

For the study design, the original authors did not adequately adjust for the reasons/indications for prescribing antiplatelet agents. Cardiovascular risk, which usually determines the choice and duration of antiplatelet therapy, was not comprehensively measured in this study. This implies that confounding by indication may still be present. The cut-off for exposure classification (≥1 year of antiplatelet therapy) was not well justified, as shorter durations of use may still have clinical relevance due to the connection between cardiovascular diseases and cancers (2). Given the risk of introducing selection or measurement bias in this approach, we suggest pre-defining an evidence-based threshold or exploring other cut-offs to avoid this issue. Additionally, the 5-year follow-up period seemed to be insufficient for detecting cancers in patients with unknown genetic risks, particularly for hepatocellular carcinoma (HCC), which usually takes longer than that (3). We suggest increasing the follow-up duration, e.g., up to 10 years, to investigate the long-term effects of antiplatelet agents. Noteworthily, while the original authors have excluded patients with non-alcoholic cirrhosis (ICD-10 K70.2 or K70.3), they did not account for its earlier stages, e.g., fibrosis or sclerosis (ICD-10 K74.0, K74.1, or K74.2). As these conditions are complications of NAFLD, they are likely associated with shorter time-to-HCC events and need adjusting to avoid confounded findings.

For the statistical analysis, while the original authors used a landmark analysis to manage the immortal time bias, this approach is not efficient based on a statistical perspective (4, 5). Landmark analysis mitigates the misclassification of immortal time but at the cost of decreased power, as it excludes patients who experience events before the landmark. Additionally, at the end of the 5-year follow-up duration, there might be a risk of covariate imbalance that could not be controlled with propensity score matching (PSM) (6). We suggest using a time-dependent approach, such as time-dependent Cox regression or g methods, for more robust findings in future observational studies (7, 8). Another issue that could introduce confounding is the subgroup analysis. The PSM only ensured the balance between the exposed and unexposed groups, not within the subgroups. Re-matching or adjusting for potential confounders should be considered to avoid biased estimates. Future investigations should also account for the competing risks in the survival analysis. For example, in this case, death by unknown causes may preclude the occurrence of cancer or the detection of cancer outcomes. To address this issue, we suggest using other methods, such as Fine–Gray subdistribution hazard model or cause-specific hazard models that include common risk factors as covariates (9, 10).

For the interpretation/reporting, subgroup analysis—if used—should be thoroughly reported and interpreted. Although subgroup/stratified results from observational studies are usually not golden evidence that warrants changes in clinical settings (11), they still implicate insights to both expert and non-expert readers. Over conclusion or misinterpretation based on these findings can easily mislead the general readers and stimulate unconfirmed practices. A well-known example of problems with subgroup analysis, although not necessarily relevant to this study, is the ISIS-2 trial, where the benefits of aspirin for acute myocardial infarction could be seen in all astrological birth signs except for Libra or Gemini (12). In this study of Anson et al., the authors did not report the p-values for interaction (1), which are needed to determine whether the differences between subgroups were highly due to chance. Thus, any interpretations implying a difference between males/females or older/younger people were likely overstating and should be treated with caution.
